# An Overview of Different Vitamin D Compounds in the Setting of Adiposity

**DOI:** 10.3390/nu16020231

**Published:** 2024-01-11

**Authors:** Eva E. Spyksma, Anastasia Alexandridou, Knut Mai, Dietrich A. Volmer, Caroline S. Stokes

**Affiliations:** 1 Food and Health Research Group, Faculty of Life Sciences, Humboldt University Berlin, 14195 Berlin, Germany; eva.ellen.spyksma@student.hu-berlin.de; 2 Bioanalytical Chemistry, Department of Chemistry, Humboldt University Berlin, 12489 Berlin, Germany; alexanda@hu-berlin.de (A.A.); dietrich.volmer@hu-berlin.de (D.A.V.); 3 Department of Endocrinology & Metabolism, Charité—Universitätsmedizin Berlin, Corporate Member of Freie Universität Berlin and Humboldt-Universität zu Berlin, 10117 Berlin, Germany; knut.mai@charite.de; 4 German Centre for Cardiovascular Research (DZHK), Partner Site Berlin, 10785 Berlin, Germany; 5 German Center for Diabetes Research, 90451 Nuremberg, Germany; 6 Department of Human Nutrition, German Institute of Human Nutrition, 14558 Nuthetal, Germany; 7 Department of Molecular Toxicology, German Institute of Human Nutrition, 14558 Nuthetal, Germany

**Keywords:** C3 epimer, inflammation, metabolic health, obesity, overweight, 25-hydroxyvitamin D

## Abstract

A large body of research shows an association between higher body weight and low vitamin D status, as assessed using serum 25-hydroxyvitamin D concentrations. Vitamin D can be metabolised in adipose tissue and has been reported to influence gene expression and modulate inflammation and adipose tissue metabolism in vitro. However, the exact metabolism of vitamin D in adipose tissue is currently unknown. White adipose tissue expresses the vitamin D receptor and hydroxylase enzymes, substantially involved in vitamin D metabolism and efficacy. The distribution and concentrations of the generated vitamin D compounds in adipose tissue, however, are largely unknown. Closing this knowledge gap could help to understand whether the different vitamin D compounds have specific health effects in the setting of adiposity. This review summarises the current evidence for a role of vitamin D in adipose tissue and discusses options to accurately measure vitamin D compounds in adipose tissue using liquid chromatography tandem mass spectrometry (LC/MS-MS).

## 1. Introduction

Vitamin D is a fat-soluble vitamin that has been reported to have many health effects. The common and most well known are the beneficial effects of vitamin D on bone density and skeletal health [1,2]. Moreover, vitamin D supplementation has been reported to decrease mortality in cancer patients and ameliorate type 2 diabetes in vitamin D-deficient individuals [3]. There are several risk factors for vitamin D deficiency, such as age [4], decreased sun exposure and skin pigmentation [5], the use of certain medications and increased adiposity [6]. Recently, vitamin D has come under scrutiny in relation to COVID-19, with studies showing a relation between low vitamin D status and higher susceptibility to infection [7] and a possible beneficial effect of vitamin D supplementation in individuals with low vitamin D levels [8]. Furthermore, Karampela et al. posed that vitamin D might be the missing link between adipose tissue and chronic inflammation [9], which is supported by in vitro evidence [10,11,12,13,14]. In addition, a positive effect of vitamin D supplementation on glucose metabolism has been reported [15,16]. The latter two effects are relevant in the setting of adiposity, a state in which white adipose tissue (WAT) is increased, which can lead to low-grade chronic inflammation and support the development of (cardio)metabolic complications, such as type 2 diabetes, hypertension and dyslipidaemia [17,18]. These findings suggest a possible synergistic role for vitamin D with other nutrients and parameters related to inflammatory and immune processes and underline its importance in contributing to an overall optimal nutrient status.

Although vitamin D has been the subject of research for decades, the full picture of vitamin D’s action in the setting of adiposity is still unclear. An unexplored area is the focus on the levels of other vitamin D compounds, in addition to the current status marker 25-hydroxyvitamin D (25(OH)D). Several of these vitamin D compounds have already been under scrutiny [19,20,21], but many questions remain unanswered. The aim here is to provide a broad overview of the current evidence surrounding the different effects of vitamin D compounds, with a special focus on people with higher body weight. Even though not all people with higher body weight are metabolically unhealthy [22], a growing part of the global population is diagnosed with metabolic syndrome [23]. Interestingly, a recent study has shown the relevance of focusing on metabolic health status instead of body weight only. Soll et al. demonstrated that the beneficial effects of a weight loss intervention were stronger and longer-term in metabolically unhealthy participants than in metabolically healthy participants with higher body weight [24]. It is of great interest to understand the relation between adiposity, metabolic status and vitamin D metabolism, which might be better understood by considering all the vitamin D compounds. Ultimately, a deeper understanding of the presence and effects of different vitamin D compounds in adipose tissue could contribute to adapting vitamin D supplementation regimens for people with higher body weight and to improving metabolic health for metabolically unhealthy people with higher body weight.

A note regarding the terminology that is used throughout this review: several studies on preferred terminology emphasise that the term “obese” is generally less favoured and that “higher weight” is a preferred and more neutral term [25]. Meadows et al. and Puhl et al. acknowledge that the use of person-first terminology is not universally appreciated and comes with its own problems [26]. This review has nevertheless chosen to use “people with higher body weight” and adiposity instead of obesity. Furthermore, to clarify the weight range being discussed, the BMI of participants is mentioned when discussing findings in human studies. In addition, adequate vitamin D levels have been stated as a serum concentration of 25(OH)D > 20 ng/mL by the US Institute of Medicine [27] and as 25(OH)D > 30 ng/mL by the Endocrine Society [6]. However, there is an ongoing debate about the sufficient serum levels of 25(OH)D, and even higher levels have also been suggested for optimal health effects [28,29]. To avoid confusion, we therefore specify 25(OH)D serum levels in ng/mL, which is the current status marker [30], when discussing studies on vitamin D status or supplementation. 

### 1.1. Vitamin D Metabolism

The precursors of 25(OH)D include vitamin D_3_ (cholecalciferol), which is found in animal products, and vitamin D_2_ (ergocalciferol), which is mainly present in mushrooms [31,32]. In humans, vitamin D_3_ can also be generated in the skin by photosynthesis after UVB exposure of the precursor 7-dehydrocholesterol. Vitamin D_2_ and vitamin D_3_ follow the same metabolic pathway. In this review, the term vitamin D is used when vitamin D_2_ or D_3_ could be used interchangeably. The conversion of vitamin D to its different compounds is achieved through the action of cytochrome P450 enzymes, for example, vitamin D 25-hydroxylases (CYP2R1, CYP27A1 and CYP2J2), 1α-hydroxylase (CYP27B1) and the aforementioned CYP24A1. The activity of these enzymes is partially dependent on magnesium as a cofactor [33,34]. Hydroxylase enzymes are mainly expressed in the liver and kidney, as well as in other tissues and cell types, such as testes, blood cells, immune cells and adipose tissue [30]. Vitamin D is converted in the liver (25 hydroxylases) and kidneys (1α hydroxylases) into bioactive 1,25-dihydroxyvitamin D (1,25(OH)_2_D). Vitamin D is catabolised by 1,25-dihydroxyvitamin D hydroxylase (CYP24A1) to 24,25-dihydroxyvitamin D (24,25(OH)_2_D) or 1,24,25-trihydroxyvitamin D (1,24,25(OH)_3_D) [30]. In circulation, vitamin D compounds are bound to the vitamin D binding protein (DBP) and albumin. 

**Figure 1:**
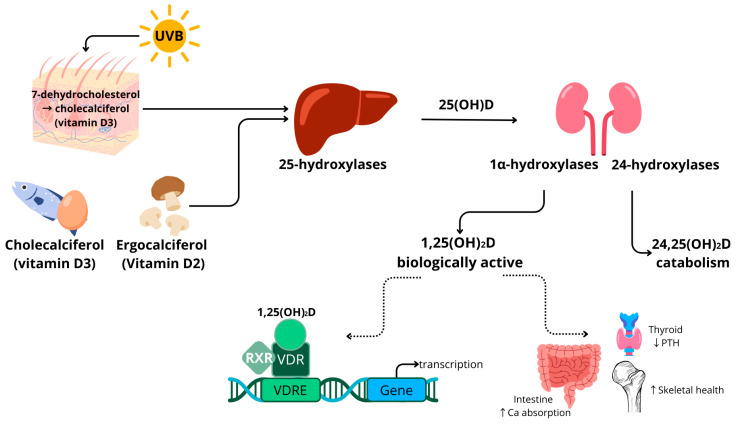
Schematic overview of vitamin D metabolism in humans. ↑ indicates an increase, ↓ indicates a decrease. Abbreviations: ultraviolet light short wavelength (UVB); calcidiol (25(OH)D); calcitriol (1,25(OH)_2_D); retinoid X receptor (RXR); vitamin D receptor (VDR); vitamin D response element (VDRE); parathyroid hormone (PTH); calcium (Ca).

A schematic overview of vitamin D metabolism is provided in Figure 1. Vitamin D binds to the vitamin D receptor (VDR), which is expressed in almost all human tissues, including adipose tissue [35]. After binding, the VDR forms a complex with the retinoid X receptor (RXR). This complex is transferred to the nucleus, where it acts as a transcription factor. Many genes have a vitamin D response element (VDRE) in their promotor regions. Target genes are related to inflammatory processes and cell differentiation, in addition to bone and calcium metabolism [36]. The transcription regulation by vitamin D is reported to be highly tissue-specific.

Vitamin D metabolism is tightly regulated by parathyroid hormone (PTH) concentrations and is linked to calcium levels [37]. Hypocalcaemia triggers the release of PTH, which, in turn, stimulates the activation of 25(OH)D to 1,25(OH)_2_D in the kidneys by CYP27B1. The compound 1,25(OH)_2_D increases intestinal calcium absorption, contributing to the normalisation of serum calcium levels and skeletal health, which is a major common health effect of vitamin D [1]. 

In addition to the metabolic pathways described above, a C-3 epimerisation pathway occurs in the liver by 3-epimerases as described by Al-Zohily et al. [38]. This results in the C-3α vitamin D compound, which has lower biological activity and less calcaemic effects. Epimers and their possible clinical relevance are discussed in more detail in the section on the specific health effects of C-3α vitamin D compounds. 

### 1.2. Vitamin D in Adiposity

Two meta-analyses demonstrated a significantly higher prevalence of low vitamin D levels (serum 25(OH)D < 30 ng/mL) in adults and children with higher body weight [39,40]. Currently, the major hypotheses for this association are the sequestration of fat-soluble vitamin D in an increased amount of adipose tissue in people with elevated body fat mass [41] and the volumetric distribution of vitamin D over a larger amount of body mass [42]. Logically, this leads to the hypothesis that weight loss could contribute to achieving vitamin D sufficiency in people with higher body weight. However, the findings are currently inconclusive. Blum et al. showed a correlation between serum vitamin D_3_ and WAT vitamin D_3_ levels [43]. They measured a mean vitamin D_3_ content in WAT of 102.8 ± 42 nmol/kg in people with a mean body mass index (BMI) of 50.6 kg/m^2^. This means that serum vitamin D could significantly improve upon weight loss if all vitamin D is released from adipose tissue. However, Himbert et al. reviewed the effect of surgery and diet-induced weight loss on serum 25(OH)D concentrations, and they concluded that weight loss did increase serum 25(OH)D levels but that this is often not enough to reach sufficient vitamin D concentrations [44]. Patients often need to be supplemented with additional vitamin D [45,46], meaning that the release of vitamin D from adipose tissue is not enough to replete individuals. This might be partially ascribed to decreased vitamin D reabsorption in the case of gastrectomy; nevertheless, a more detailed understanding of the metabolic fate of vitamin D in adipose tissue is warranted. 

The expression and protein secretion of the hydroxylase enzymes involved in the conversion of vitamin D have been reported in many tissues, including WAT [47]. Human adipocytes were shown to express CYP27B1, as well as CYP24A1 [48]. The expression of the enzymes CYP2R1 and CYP2J2 was also shown in human adipose tissue [49]. Jonas et al. demonstrated an increased CYP27B1 expression in WAT of people with higher body weight (BMI > 40 kg/m^2^) [50]. In another study, however, the expression of CYP2J2 and CYP27B1 was decreased in women with higher body weight (BMI > 35 kg/m^2^) [49]. These contradicting results illustrate that more research is necessary to understand the link between vitamin D metabolism and adipose tissue. However, hypotheses do exist; for example, the expression of CYP24A1 in adipose tissue could lead to an increased degradation of 1,25(OH)_2_D in people with higher body weight. This effect could be aggravated by the concomitant downregulation of CYP2R1, another 25-hydroxylase, which was seen in a mouse model of obesity [51]. Such changes in hydroxylase enzyme expression could be a partial explanation for the decreased vitamin D status in people with higher body weight and underline the link between adipose tissue and vitamin D metabolism. 

**Figure 2:**
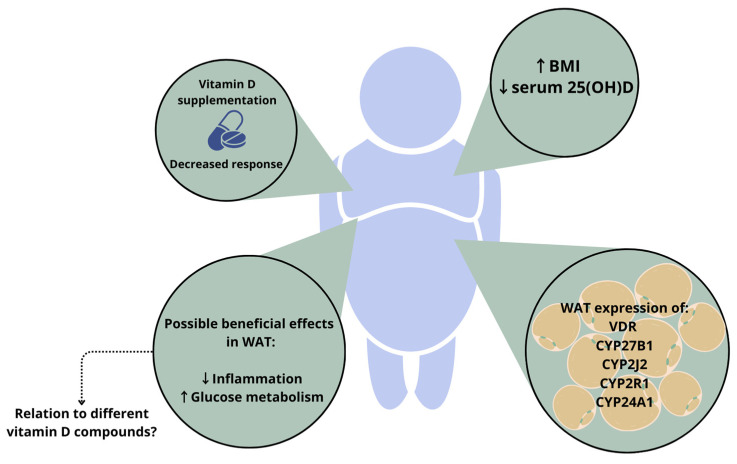
Depiction of the relation between vitamin D status and white adipose tissue (WAT) mass in people with higher body weight. The response to vitamin D supplementation is altered in people with higher body weight, and an increased BMI is associated with lower serum 25(OH)D levels. In addition, white adipocytes express the VDR and hydroxylase enzymes. Vitamin D can have a beneficial effect on processes in WAT; however, it is unknown whether these effects are mediated by specific vitamin D compounds. ↑ indicates an increase, ↓ indicates a decrease. Abbreviations: BMI, body mass index; VDR, vitamin D receptor; WAT, white adipose tissue; 25(OH)D, 25-hydroxyvitamin D.

In addition to hydroxylase enzymes, adipocytes express the VDR [48,49], meaning that vitamin D compounds binding the receptor can have a direct local effect on gene transcription in adipose tissue. Adipose VDR is reported to be increased in obesity [50]. A genomic study showed that the rs3782905 single-nucleotide polymorphism (SNP) in the VDR gene is associated with differences in adiposity [52], which indicates a relation between vitamin D signalling and the development of adipose tissue. However, the impact of different VDR genotypes on the development of adiposity is not supported by all data [53]. Together, these findings on hydroxylase enzymes and VDR expression indicate once again that vitamin D metabolism is linked to adipose tissue mass. The relations between WAT and vitamin D metabolism in people with higher body weight are depicted in Figure 2. 

Body weight has also been reported to influence the response to vitamin D supplementation [54]. A recent meta-analysis reported that vitamin D_3_ supplementation is more effective than vitamin D_2_ at raising serum 25(OH)D concentrations. However, the authors reported that baseline status needs to be considered and that this seems to be less relevant for people with higher body weight, since having a BMI > 25 k/m^2^ reduced the difference in response to vitamin D_3_ and vitamin D_2_ supplementation [55]. Moreover, a large vitamin D supplementation trial demonstrated that BMI modified the effect of vitamin D supplementation (2000 IU/day) on serum 25(OH)D levels. In a post hoc analysis, the participants were stratified in BMI categories, and the increase in serum 25(OH)D was observed to be blunted in higher BMI classes. Furthermore, altered vitamin D metabolism in people with higher body weight (BMI > 30 kg/m^2^) was reported in a randomised controlled trial (RCT) in which the effectiveness of supplementation with vitamin D_3_ was compared to that of 25(OH)D_3_ [56]. Compared to 25(OH)D_3_, an extra 30 µg/week (1200 IU) of vitamin D_3_ was required to achieve comparable serum concentrations of 25(OH)D in people with higher body weight. This effect was not seen in those with a lower BMI. Several reviews have described a relation between vitamin D status, body weight and health outcomes [57,58,59], and they have concluded that vitamin D might influence adipose tissue metabolism via so far unknown pathways and that vitamin D supplementation could be beneficial for people with higher body weight. However, the results described above show that vitamin D supplementation needs to be tailored to each individual. 

In this context, it is also important to consider the differences between the different types of adipose tissue. Visceral adipose tissue (VAT) expansion seems to have a more unfavourable effect on metabolic health than an increase in subcutaneous adipose tissue (SAT) [60,61]. Cordeiro et al. [62] summarised the studies that assessed the relation between vitamin D status and SAT and VAT. Due to the lack of sufficient data, no final conclusions could be drawn regarding tissue-specific differences in vitamin D metabolism and VDR expression. However, a small body of data is currently available. Jonas et al. did not show any differences in VDR and CYP27B1 expression in SAT and VAT [50]. Wamberg et al. reported differences in decreased CYP27A1 expression in SAT compared to VAT [49]. Biopsy studies revealed higher vitamin D_3_ concentrations in VAT than in SAT [63]. Accordingly, the increase in serum 25(OH)D in response to vitamin D supplementation has been correlated with VAT but not with SAT mass [64]. In summary, these data indicate that there are differences between vitamin D metabolism in SAT and VAT; however, more research on the association between vitamin D compound levels and vitamin D metabolism is needed to fully understand the specific effects of vitamin D on different adipose tissue depots.

Of note, the rapid expansion of WAT in people with higher body weight can lead to an inflammatory response in adipocytes and adipose tissue macrophages [17]. These changes in adipose tissue function induce an increase in insulin resistance, which is a marker of a metabolically unhealthy phenotype in people with higher body weight [23,65]. Considering the reported association of vitamin D with inflammation [66,67] and glucose metabolism [15,16], differences in vitamin D availability or action in adipose tissue could play a crucial role in this context. Vitamin D can locally affect adipose tissue via the adipose VDR and is metabolised within adipose tissue by hydroxylase enzymes. Therefore, it is of importance to know more about the levels of vitamin D compounds and to determine whether they have specific health effects within adipose tissue. 

## 2. Relevance of Different Vitamin D Compounds

### 2.1. Free Vitamin D Compound

The majority of vitamin D is bound to the vitamin D binding protein (DBP). Megalin/cubulin receptors enable the entry of the vitamin D-DBP complex into cells [68]. The proportion of free vitamin D is a very small fraction in comparison to that of protein-bound vitamin D, but it can move freely over cell membranes [69]. According to the free hormone hypothesis, this makes free vitamin D the most biologically active vitamin D compound [69,70,71], and, thus, this would make it very relevant in relation to the health effects of vitamin D. 

Schwartz et al. performed a large cross-sectional study, in which they measured total serum 25(OH)D and free 25(OH)D levels in people with liver cirrhosis, pregnant women and healthy controls [72]. They observed that the levels of free 25(OH)D were significantly higher in people with liver disease, most likely due to a decreased capacity to produce DBP. A study by Shieh et al. offered in vivo indicators suggesting that free vitamin D plays a physiologically relevant role in humans [73]. They showed that, during the early stages of vitamin D repletion in deficient participants, the association between free vitamin D and 24,25(OH)_2_D and PTH in serum was stronger than the association between these compounds and total serum 25(OH)D. This indicates that free vitamin D was converted to 1,25(OH)_2_D and then inactivated in the kidneys. Thus, the free form was taken up into the parathyroid and kidney cells, regardless of the megalin/cubilin expression in these cell types, where it was converted to other compounds and exerted biological effects. 

A bariatric surgery study showed that weight loss led to differential changes in the serum levels of free 25(OH)D and total 25(OH)D [74]. The serum levels of total 25(OH)D did not significantly increase when assessed one-year post-surgery, whereas a rise in the serum levels of free 25(OH)D could be observed. This could indicate that free vitamin D is the main storage compound of vitamin D in adipose tissue. However, it should be noted that there are several other mechanisms that could explain this differential effect, such as changes in serum protein levels or the expression of the vitamin D binding protein after weight loss. In a study comparing lean women to women with higher body weight (BMI = 39.1 ± 4.6 kg/m^2^), DBP levels were higher and free vitamin D levels were lower in the latter group [75]. The increased DBP levels could potentially have an effect on the availability of free vitamin D in tissues. Of note, the free vitamin D levels in this study were calculated rather than quantified with immunoassays or mass spectrometry. However, Rivera-Paredez et al. observed no differences between free vitamin D and total serum 25(OH)D when comparing associations with markers of metabolic health in Mexican adults [76]. However, the participants of this study had a BMI between 24 and 30 kg/m^2^, which is considerably less than in the previously discussed studies. This could be an indication that adiposity significantly alters the role and presence of different vitamin D compounds. In conclusion, there are some findings that indicate the differential effects of adiposity and weight loss on free and bound or total vitamin D levels; however, more research is needed to clearly define the physiological relevance of free vitamin D in the context of adiposity.

### 2.2. 1,25-Dihydroxyvitamin D (1,25(OH)_2_D)

The 1,25(OH)_2_D compound is generally considered the active form due to its high affinity for the VDR [77]. This section highlights two health effects of 1,25(OH)_2_D that are highly relevant in the setting of adiposity: firstly, the relation to inflammation and, secondly, the effect on adipokine secretion. 

With regard to the anti-inflammatory effects of 1,25(OH)_2_D, NFkB signalling has been implicated as a potential mechanism, since vitamin D has been reported to decrease NFkB expression [12] and to block NFkB translocation to the nucleus [13,78,79], thus decreasing the transcription of pro-inflammatory cytokines. Even though these findings directly link 1,25(OH)_2_D to decreased inflammation in vitro, there are contradicting reports of the effects of vitamin D supplementation on inflammatory markers in vivo. Wamberg et al. [80] observed a diminishing effect on inflammation in an adipocyte cell line treated with 1,25(OH)_2_D_3_. However, this effect was not reproduced in primary human adipocytes, and it was not reflected in circulating inflammatory markers in vivo [80]. A cross-sectional study did show a negative correlation between serum 25(OH)D levels and the serum levels of IL-6 and TNF-α in women with obesity (mean BMI of 43.6 ± 4.3 kg/m^2^) [81]. This is confirmed in other cross-sectional studies that report a relation between vitamin D status and the levels of inflammatory markers in blood [82]. These studies reported the serum levels of 25(OH)D but not those of 1,25(OH)_2_D. Nevertheless, 25(OH)D is a precursor of 1,25(OH)_2_D, and it would be of great interest to further investigate, for example, the effect of vitamin D supplementation on inflammatory markers within adipose tissue because this is a major storage organ of vitamin D [83]. 

Adipokines are messenger molecules produced by adipocytes. They affect energy metabolism, insulin resistance, inflammation and blood pressure and are strongly related to metabolic health [84,85]. Two well-known examples are leptin and adiponectin. Leptin is known as the satiety hormone, which, in healthy conditions, supresses food intake, regulates energy metabolism and acts as a negative feedback loop upon adipose tissue accumulation [86,87]. However, in people with higher body weight, leptin resistance frequently occurs [88,89,90]. Adiponectin is an adipose-tissue-derived hormone with health-promoting effects. It has been reported to increase insulin sensitivity and have anti-inflammatory properties [91]. Adiponectin levels are often decreased in people with increased body fat [92]. In two in vitro studies, 1,25(OH)_2_D_3_ was reported to decrease leptin production in primary human adipocytes [12,93]. This is supported by several human studies, although these assessed 25(OH)D instead of 1,25(OH)_2_D. Cross-sectional studies showed a negative association between low serum 25(OH)D and high leptin levels [94,95,96]. In a study in patients with prediabetes, the association was present after adjustment for BMI, supporting the notion that vitamin D could have direct effects on leptin [97]. However, negative associations between leptin and 25(OH)D have also been reported [95,98]. 

The effects of 1,25(OH)_2_D exposure on adiponectin have also been reported, e.g., decreased adiponectin production in human primary adipocytes [99]. Accordingly, a negative association between adiponectin levels and serum 25(OH)D was observed in humans [97]. However, this effect was only reported in a subgroup of participants with a BMI > 25 kg/m^2^. In another study, no association between serum 25(OH)D and adiponectin was reported [100]. And yet another study found a positive correlation between 25(OH)D and adiponectin levels [96]. Two trials did not show an effect of vitamin D on adipokine levels [101,102]. O’Sullivan et al. found no effect of 600 IU/day supplementation during a four-week period, but the sample consisted mostly of participants with baseline vitamin D sufficiency. Upon further analysis, it was found that a subsample of participants who were vitamin D-deficient had higher serum adiponectin levels at baseline and were more responsive to vitamin D supplementation. 

Together, these findings illustrate that 1,25(OH)_2_D can regulate important processes in human adipose tissue. This makes it of interest to assess the levels of this vitamin D compound in the adipose tissue or serum of people with higher body weight.

### 2.3. 24,25-Dihydroxyvitamin D (24,25(OH)_2_D)

The 24,25(OH)_2_D compound is a clearance product in the vitamin D metabolic cascade. The ratio of 24,25(OH)_2_D to 25(OH)D has been suggested to be an alternative marker of vitamin D status [103]. This ratio takes vitamin D metabolism and feedback loops into account and is not dependent on the level of DBP [104,105]. When assessing other vitamin D compounds, Binkley et al. described the predictive value of 24,25(OH)_2_D for the response to vitamin D supplementation [20]. In this study, the ratio of 24,25(OH)_2_D/25(OH)D did not predict the response to vitamin D supplementation, contradicting earlier suggestions of 24,25(OH)_2_D/25(OH)D being an improved status marker [103,106]. However, the sample size was relatively small (*n* = 62) and consisted of postmenopausal women only. These data indicate that the measurement of 24,25(OH)_2_D could have an additional clinical relevance to measuring only serum 25(OH)D [106]. Furthermore, 24,25(OH)_2_D has its own receptor, the membrane receptor family with sequence similarity 57 (FAM57B2) [107], and it has been reported to have biological effects, e.g., in relation to bone fracture healing and decreased tumorigenesis [108]. However, the role in inflammation, insulin resistance and adipose tissue function, as well as the distribution in different adipose tissue compartments, is currently not fully understood. 

### 2.4. C-3 Epimers of Vitamin D Compounds

In vitamin D metabolism, a parallel C-3 epimerisation pathway introduces additional vitamin D compounds. The most important of these is the C-3α isomer of 25(OH)D, which results from the reversal of the stereochemical configuration of the –OH group at C-3 (3β→3α). It is important to measure this compound for two reasons. Firstly, the 3α epimer has been reported to lead to the overestimation of vitamin D status with current detection methods, such as immunoassays and MS assays, if it is not properly separated [109]. The extent of this additional contribution depends on physiological and pathological conditions and age; e.g., 3α levels are naturally higher in newborns, especially in those born prematurely [110,111]. Secondly, the 3α epimer has demonstrated biological activity of its own, e.g., anti-proliferative effects, increased phospholipid production [38,111] and cardiovascular function [112]. The latter was concluded in a cohort study that showed an association of aerobic capacity with the 3α epimer of vitamin D in patients with chronic kidney disease. 

With respect to body composition, a negative correlation was reported for serum 25(OH)D concentrations and fat mass in infants at 12 months in a vitamin D supplementation trial [113]. The authors reported that serum 25(OH)D was a predictor of lean mass and fat mass in regression models. This correlation was only present at the age of three months for 3α-25(OH)D, even though 3β-25(OH)D was associated with all time points. In a Thai national health survey, the relative amount of 3α-25(OH)D_3_ was associated with age, sex and living conditions [114]. Males and people living in rural areas had higher relative concentrations of serum 3α-25(OH)D_3_. The survey observed a negative correlation between BMI and 3β-25(OH)D, but not 3α-25(OH)D. Another study that included women with polycystic ovary syndrome (PCOS) and healthy controls did not observe differences between the two groups in 3β and 3α-25(OH)D_3_ [115]. A recent clinical study investigated the levels of 1,25(OH)_2_D_3_ and 3α-25(OH)D_3_ in participants with normal versus higher body weight [116]. No association differences between serum vitamin D compounds and body weight were found. Furthermore, none of the compounds were associated with inflammatory or metabolic health markers. Overall, the biological actions of the 3α epimer require more research.

## 3. Measuring Vitamin D Compounds in Human Adipose Tissue with LC-MS/MS

Vitamin D compounds are usually measured using either immunoassays or mass spectrometry (MS). While immunoassays are automated and fast and the cost of analysis is low in comparison to other instrumental techniques [117], they sometimes lack selectivity and do not provide the possibility of analysing multiple vitamin D compounds at the same time [118,119]. MS assays are currently the “gold standard” method [21] because they provide measurements of multiple vitamin D compounds simultaneously, with high selectivity and very low limits of quantification (LOQ). Sample preparation, however, is more laborious, especially when analysing adipose tissue. For readers interested in a detailed description of mass spectrometric methods for vitamin D compounds, not limited to adipose tissue samples, we refer to other reviews on this topic [120,121]. The benefits of MS assays compared to established immunoassays have also been described previously [122,123,124]. Table 1 summarises several important studies on adipose tissue analysis using MS, along with the required sample quantity, steps of sample preparation, measured compounds, LOQ or limit of detection (LOD) and recovery of the sample preparation protocol. The protocols mentioned in the table are described in brief in this chapter. 

Generally, sample preparation protocols for adipose tissue samples for subsequent mass spectrometry analysis are more complicated, time-consuming and expensive than those for conventional blood sample analysis, which usually consist of some or all of the following steps: protein precipitation, which separates analytes from binding proteins and removes proteins from the matrix, and saponification, which removes lipids. This can be achieved by adding potassium hydroxide to the sample and leaving it overnight [125]. Shorter saponification reaction times can be achieved at higher temperatures, but a careful balance has to be found for the thermally instable vitamin D [121]. Usually, these steps are followed by liquid–liquid extraction (LLE) and/or solid-phase extraction (SPE). This is sometimes also followed by a chemical derivatisation step; this enhances ionisation sensitivity for vitamin D compounds, which exhibit low ionisation efficiency [122,126].

**Table 1:** Overview of sample preparation protocols used to measure vitamin D compounds in adipose tissue.

Author, Year	MS, Ionisation	Sample Weight (g)	Sample Preparation	Measured Compounds	LOD LOQ	Recovery (R%)
Blum et al., 2008 [43]	LC-MS APCI	0.2–0.25	Homogenisation Saponification LLE SPE	D_3_	-	72%
Höller et al., 2010 [127]	LC-MS APCI	0.5	Homogenisation Protein precipitation SPE C-18	25(OH)D_3_	LOD 5 ng/g	-
Beckman et al., 2013 [63]	LC DAD	-	Saponification LLE SPE Preparative HPLC	D_2_ D_3_	-	98.5 ± 5%
Piccolo et al., 2013 [128]	LC-MS/MS APCI	0.1–0.5	Homogenisation SPE Filtration	25(OH)D_3_		
Lipkie et al., 2013 [129]	LC-MS/MS ESI	-	Homogenisation LLE SPE PTAD derivatisation	25(OH)D_2_ 25(OH)D_3_ D_2_ D_3_		
Malmberg et al., 2014 [130]	SIMS-TOF			D_3_ 25(OH)D_3_ 1,25(OH)_2_D_3_	-	-
Burild et al., 2014 [131] Didriksen et al., 2015 [132] Martinaityte et al., 2017 [133]	LC-MS/MS ESI	0.2–1.0	Saponification LLE SPE PTAD derivatisation	D_3_ 25(OH)D_3_	LOQ < 0.1 ng/g	-
Bonnet et al., 2019 [134] Bonnet et al., 2021 [135]	LC-MS/MS ESI	0.05	Homogenisation LLE SPE Amplifex derivatisation	D_3_ 25(OH)D_3_ 1,25(OH)_2_D_3_	-	-
Best et al., 2021 [136]	LC-MS/MS	0.01	Saponification LLE PTAD derivatisation	D_3_ 25(OH)D_3_	-	-

As mentioned above, adipose tissue is a challenging matrix because of its complex lipid composition, from which vitamin D compounds, which are themselves lipids, have to be extracted. As a result, the protocols in Table 1 are very laborious in comparison to other matrices [121] and still do not always guarantee the successful detection of vitamin D compounds. This was shown by Höller et al., who measured 25(OH)D_3_ in skin, kidney, liver and muscle tissue, and found that the levels of the compounds were lower than the lower limits of quantification (LLOQ) of the method in adipose tissue [127]. Derivatisation can improve LOQ, as demonstrated by Lipkie et al., who measured 25(OH)D_2_, 25(OH)D_3_, vitamin D_2_ and D_3_ in the liver, muscle and epididymal adipose tissue of rats receiving a vitamin D-enriched diet [129]. The authors used LLE, followed by SPE and derivatisation with 4-phenyl-1,2,4-triazole-3,5-dione (PTAD). LOD was 0.1 ng/mL for all investigated vitamin D compounds. The reported accuracies of quantification were based on National Institute of Standards and Technology (NIST) reference materials, giving 78% and 129% for 25(OH)D_2_ and 25(OH)D_3_, respectively, which did not meet the standards outlined by the US Food and Drug Administration (FDA) [137]. Bonnet et al. developed an LC-MS/MS method using high-resolution orbitrap MS and measured vitamin D_3_, 25(OH)D_3_ and 1,25(OH)_2_D_3_ in the adipose tissue of mice [134,135] using Lipkie et al.’s [129] sample preparation protocol. The authors implemented Amplifex instead of PTAD for lower LOQs of 0.78, 0.19 and 0.02 ng/mL for cholecalciferol, 25(OH)D_3_ and 1,25(OH)_2_D_3_. Precision (RE%) and accuracy (CV%) were <15%, thus complying with the FDA guidelines [137]. Of note, there are other derivatisation options for vitamin D compounds, developed for serum or plasma-based assays equally applied to extracts from tissue samples [126]. 

The chromatographic separation of vitamin D compounds is usually performed using reversed-phase C-18 stationary phases. Two exceptions were developed by Blum et al. [43] and Picollo et al. [128], who used C-30 stationary phase chemistry, which can exhibit increased selectivity and retention over C-18 columns for some compounds [138]. Ionisation is almost always achieved using either atmospheric pressure chemical ionisation (APCI) or electrospray ionisation (ESI) in positive ion mode prior to MS detection. An exception was reported by Beckman et al., who used diode-array detection (HPLC-DAD) [63]. The authors reported the average recovery of vitamin D_2_ and D_3_ to be 98.5% ± 5%. Vitamin D_2_ was not detectable in most samples. However, vitamin D_3_ was measured in both subcutaneous and visceral adipose tissue, with VAT showing greater affinity for vitamin D_3_ storage. No LOD or LOQ was reported. Burild et al. utilised overnight saponification at room temperature, two steps of LLE and PTAD derivatisation. LOQ was reported to be <0.1 ng/g, and accuracies were 84–96% and 113–114% for vitamin D_3_ and 25(OH)D_3_, respectively; AOAC validation criteria were met [131]. Didriksen et al. and Martinaityte et al. studied human adipose tissue samples following the same protocol [132,133]. Best et al. described a protocol with a saponification step performed at 95 °C [136]. Unfortunately, no recovery or LOQ or LOD values were reported for the determination of vitamin D compounds in adipose tissue. 

Finally, Malmberg et al. reported an imaging mass spectrometry protocol for adipose tissue using secondary ion mass spectrometry (SIMS) [130], which required an entirely different sample preparation protocol, as the spatial integrity of vitamin D_3_ in the tissue had to be maintained. The authors investigated the spatial distribution of vitamin D_3_, 25(OH)D_3_ and 1,25(OH)_2_D_3_ in adipose tissue [130]. The protocol consisted of freezing the adipose tissue samples under high pressure, as well as freeze-fracturing them. Vitamin D_3_, 25(OH)D_3_, and 1,25(OH)_2_D_3_ were found in the adipocyte lipid droplet. The observed signals for 25(OH)D_3_ and 1,25(OH)_2_D_3_ were very low, making a comparison of the distributions for the different compounds challenging. 

## 4. Conclusions

This review explores the evidence base for the associations of different vitamin D compounds with health markers and specific biological effects of vitamin D compounds, specifically in relation to increased body weight and different adipose tissue components. Furthermore, to the best of our knowledge, this is the first review to include an overview of LC-MS/MS sample preparation methods to accurately and simultaneously measure different vitamin D compounds in human adipose tissue. 

Vitamin D compounds, such as 1,25(OH)_2_D, free vitamin D and the 3α epimer of 25(OH)D, in addition to the status marker 25(OH)D, have been reported to have various extra-skeletal health effects that are highly relevant in the setting of increased adiposity. Investigating vitamin D concentrations in human adipose tissue might shed further light on the role of vitamin D with regard to adipose tissue inflammation. Vitamin D has also been shown to affect adipokine levels, which could be an important approach to modulate metabolic health. Vitamin D, though not the only factor influencing the development of inflammation in adipose tissue, likely plays an important role, including in adipokine synthesis and adipocyte biology in general. Thus, a comprehensive approach to these processes is needed in order to understand the impact of individual factors, such as vitamin D. 

While the expression of hydroxylase enzymes and the VDR in WAT has been demonstrated [49,50], it is not clear how these enzymes affect the concentrations of different vitamin D compounds in adipose tissue and how this affects health in people with higher body weight. More studies focusing on multiple vitamin D compounds and their concentrations in SAT and VAT are required to shed further light on this topic. Preliminary studies indicate that vitamin D compounds (e.g., serum free 25(OH)D_3_ and serum total 25(OH)D) show differential responses to weight loss and that vitamin D compounds demonstrate different associations with sex or health conditions. No differential associations have been observed with inflammatory markers, and, to date, no predictive effects of compound ratios on vitamin D supplementation have been demonstrated. However, further research in this area is needed.
